# Evaluation of an Innovative Bleeding Cricothyrotomy Model

**DOI:** 10.7759/cureus.3327

**Published:** 2018-09-18

**Authors:** Kate E Hughes, David Biffar, Eze O Ahanonu, Thomas M Cahir, Allan Hamilton, John C Sakles

**Affiliations:** 1 Emergency Medicine, University of Arizona, Tucson, USA; 2 Health Sciences, University of Arizona, Tucson, USA; 3 Electrical and Computer Engineering, University of Arizona, Tucson, USA; 4 Surgery, University of Arizona, Tucson, USA

**Keywords:** airway, emergency, simulation, bleeding, cricothyrotomy, resident, education

## Abstract

Objectives

Emergency medicine (EM) residents are required to perform a cricothyrotomy during training as per the Accreditation Council for Graduate Medical Education (ACGME) guidelines. Cricothyrotomy is a rare procedure, comprising 0.45% of emergency department airway management procedures. Procedural competence in utilizing a realistic trainer is of utmost importance. We have developed a cricothyrotomy trainer using a fused deposition modeling (FDM) three-dimensional (3D) printer and innovative bleeding tissue to enhance fidelity. We aim to evaluate the trainer’s realism.

Methods

Implementation occurred during a difficult airway educational lab for EM residents in April 2018. Participants completed anonymous written surveys after performing a cricothyrotomy on the trainer. The survey evaluated the realism of the trainer and compared it to other available models by utilizing five-point visual analog scales (VAS). The participants rated their comfort level in performing the procedure pre- and post-educational lab on a five-point VAS. Demographic data included postgraduate year, prior clinical cricothyrotomy experience as a primary operator versus as an assistant, and previous trainer experience. The survey included open-response suggestions for trainer improvement.

Results

Forty-three EM residents completed the survey (82.7%, 43/52). The mean realism rating of the trainer was 3.81 (95% CI = 3.54-4.1). The participants reported previous training on cadaver (62.8%, 27/43), porcine (46.5%, 20/43), and manikin (67.4%, 29/43) models prior to using this trainer. The bleeding cricothyrotomy trainer was rated higher than other models (4.45, 95% CI = 4.28-4.63). Participants noted improved comfort with performing the cricothyrotomy after the educational lab (average improvement of 1.23±0.75). Participants specifically commented on the realism of the bleeding and skin texture; however, they also recommended a reduction in the size of the cricothyroid membrane space.

Conclusion

The innovative bleeding cricothyrotomy trainer has greater fidelity and reported superiority when compared to other commonly used nonbleeding models. This trainer provides a more advanced platform to teach an infrequent yet critical procedural skill to emergency medicine residents.

## Introduction

Cricothyrotomy is a life-saving procedure that is performed by emergency medicine (EM) physicians in rare 'cannot intubate, cannot oxygenate' scenarios [1]. With a prevalence noted to be 0.45% of the emergency department's airway management procedures, cricothyrotomy is a high-risk, low-frequency procedure [2-3]. Most emergency physicians will graduate from residency without having performed a cricothyrotomy on a patient [4]. EM residents are required to perform three cricothyrotomies during their training, either live or simulated, per the EM Residency Review Committee (RRC) guidelines [5]. Thus, having a realistic task trainer to teach EM residents this life-saving procedure is of utmost importance in order to ensure competence. 

Cadaver and porcine training is noted to be superior to simulation training for procedural fidelity [6-7]. Cost and supply restraints limit the training programs’ ability to use cadavers, especially when training numerous residents. A learner may require up to five training experiences in order to successfully perform a cricothyrotomy within an adequate time limit - thus, durability and reusability are of significant concern [1]. Several other trainers are utilized for education, including porcine models, simulation manikins, and low-fidelity trainers. Although these trainers may be low-cost, the realism of the cricothyrotomy is hindered by a lack of bleeding and easy anatomy visualization. 

We developed a reusable cricothyrotomy trainer with an innovative bleeding tissue to address this gap and enhance procedural realism. The aim of this technical report was to evaluate the structural and functional fidelity of this novel task trainer.

## Materials and methods

Technical report

**Figure 1:**
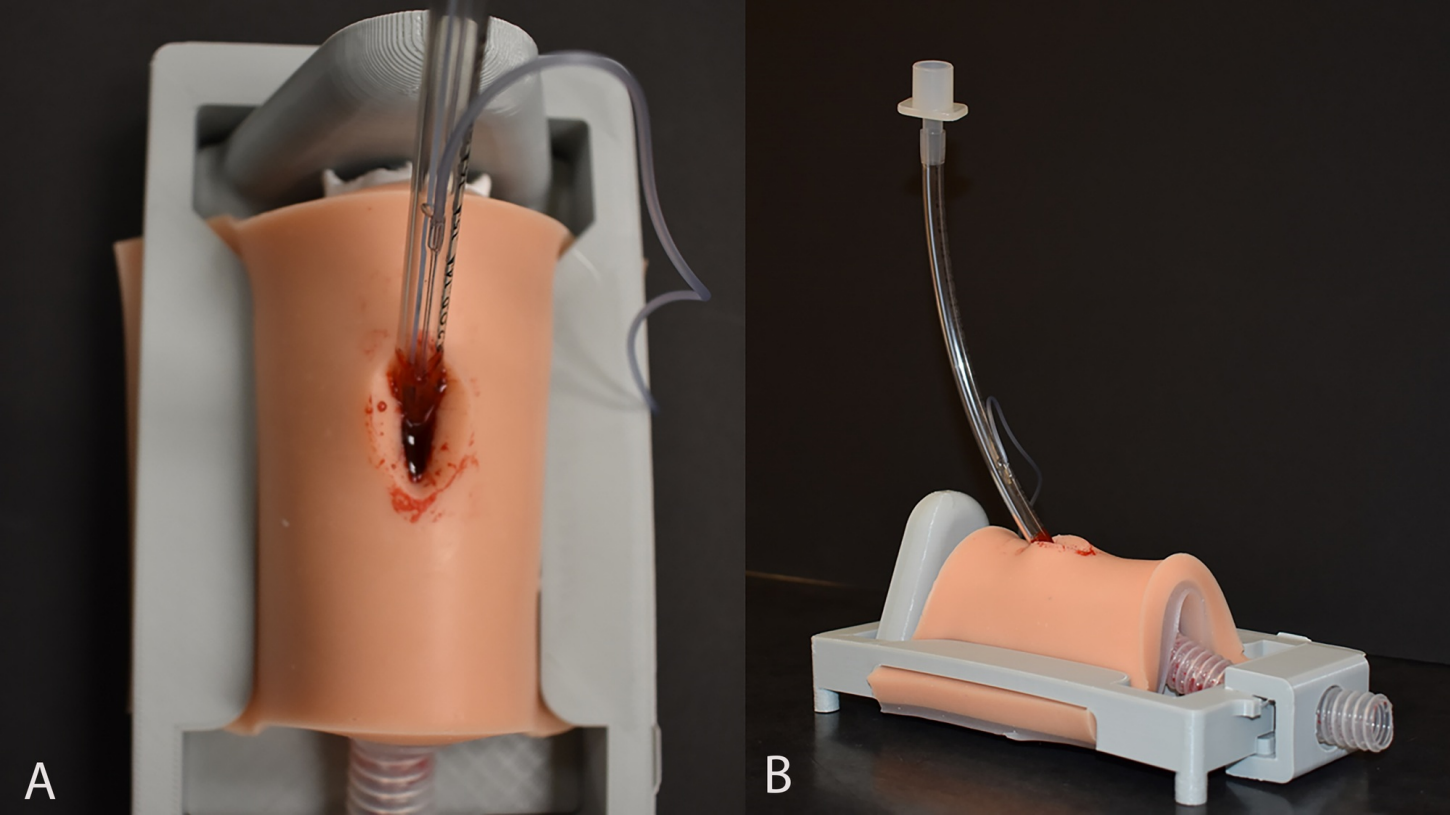
A bleeding cricothyrotomy model with an endotracheal tube A. Birds-eye view of model, showing obscured visualization of the cricothyroid membrane due to artificial blood B. Lateral view of model

The trainer comprises three sections: a three-dimensional (3D) printed housing apparatus, a 3D printed anatomical trachea, and a two-layer silicone pad that accommodates artificial blood (Figure 1A, 1B). The stereolithographic (STL) trachea model is an open-source model that is available online on the Airway app (Pendar Labs, Vancouver, British Columbia, Canada) [8]. A LulzBot TAZ 5 (Aleph Objects, Loveland, CO, USA) fused deposition modeling (FDM) 3D printer produced the trachea using 3.0 mm diameter polylactic acid (PLA) filament via a temperature-regulated extruder with 50-micron layer resolution. The housing apparatus was designed with a computer-aided design (CAD) software, Inventor (Autodesk, San Rafael, CA, USA), and printed in PLA on the LulzBot TAZ 5 printer. It accommodates tissue pads of varying thickness. This layered silicone pad accommodates artificial blood (PPC, Chantilly, VA, USA) and consists of two-part siliocine of both Dragon Skin and Ecoflex (Smooth-on, Macungie, PA, USA). Tissue thickness was controlled by volumetric additive deposition. 3M Durapore 1-inch surgical tape (The 3M company, Maplewood, Minnesota, U.S) created the membrane. The pad was dusted with baby powder to reduce tackiness and mimic human tissue texture. The materials cost to make a single, reusable 3D printed housing is approximately 7 USD, and the materials cost of each single-use bleeding tissue pad is about 6 USD. 

Pilot testing, participants, and demographics

The trainer was pilot-tested for fidelity by a convenience sample of three academic EM faculty physicians. All were comfortable with performing cricothyrotomy prior to use. Modifications were made after pilot-testing to increase the thickness of the bleeding tissue.

Implementation occurred during an annual difficult airway educational lab for EM residents in April 2018. There are 79 residents among two three-year EM programs and one five-year combined EM-pediatrics program. These residents train in a level I trauma center with an annual volume of 80,000 patients and a level IV trauma center with 50,000 visits annually; approximately one cricothyrotomy is performed between these centers every two years. The educational lab occurred in the simulation center. Residents attended a one-hour didactic lecture from an academic EM attending regarding the procedural technique before participating in the hands-on lab portion.

Curriculum development and outline

Participants completed anonymous written surveys after performing a cricothyrotomy on the trainer. The survey was created by a simulation-fellowship trained emergency physician and was reviewed by three emergency physicians with expertise in airway management and medical simulation. The survey rated the realism of the trainer and asked participants to compare it to previous models they have utilized with five-point visual analog scales (VAS). Participants rated their comfort level in performing the procedure before and after the lab on a five-point VAS. All participating residents were included. Demographic data included their postgraduate year (PGY), prior experience with clinical cricothyrotomies as the primary operator versus the assistant, and previous trainer experience. The survey also included open-response suggestions for trainer improvement. 

The data was entered into an Excel spreadsheet and summary statistics were drawn up. The comfort level differences between pre- and post-lab experience were compared using a paired* t* test. The institutional review board determined the study to be exempt and informed consent was waived.

## Results

Fifty-two EM residents participated in the educational lab, and 43 completed the anonymous survey (82.7%). Demographic data are summarized in Table 1. The mean realism rating of the trainer was 3.81 (95% CI = 3.54 - 4.1). The bleeding cricothyrotomy trainer was rated higher than the previously used models (mean 4.45, 95% CI = 4.28-4.63). The participants noted an improved comfort with performing a cricothyrotomy after the educational lab experience (average improvement of 1.23 ± 0.75). The initial mean comfort level was 2.49, and post-lab comfort level mean was 3.72 (p<0.0001). Participants also specifically commented on the realism of the bleeding tissue and texture of the skin. The palpation of the cricothyroid membrane space was felt to be too obvious, and several participants suggested decreasing its size. 

**Table 1:** Demographic characteristics of survey population N = 43

Characteristics	No. (%)
Postgraduate year	
1	19 (44.2)
2	14 (32.6)
3	8 (18.6)
4	1 (2.3)
5	1 (2.3)
Primary operator for procedure	1 (2.3)
Assisted with procedure	5 (11.6)
Observed procedure	8 (18.6)
Previous models used	
Cadaver	27 (62.8)
Porcine	20 (46.5)
Simulation manikin	29 (67.4)

## Discussion

As a result of the wide array of available airway devices, the need for a surgical airway is decreasing. Many graduating EM residents will have never performed a cricothyrotomy in clinical practice [4,9]. The development of a trainer that can realistically simulate the procedure is necessary to train competent EM physicians [5]. Participants felt that the bleeding cricothyrotomy trainer had a high degree of structural and functional fidelity and demonstrated superior fidelity to other models previously used. This trainer is unique in that bleeding from the incision provided more realistic obstacles in visualization for airway placement than models without this feature. This is invaluable as clinical visualization can be virtually impossible due to bleeding. 

Many other models do not offer bleeding and allow a clear visualization of the anatomy. Residents have commented on the bleeding tissue, stating that the “bleeding was very realistic” and that this is a “much better model due to the bleeding.” Cricothyrotomy requires tactile skills in the clinical setting and the majority of current trainers are unrealistic in this factor.

Cricothyrotomy is a rare procedure that is high-acuity in nature. Accordingly, the EM residents surveyed reported a comfort level of “uncomfortable” with performing the procedure prior to the educational lab. The majority of residents were novices in cricothyrotomy. After both didactic lectures and hands-on practice in the simulation lab, under the direct supervision of trained faculty instructors, residents did note that there was an improvement in their comfort to mildly comfortable. It is important to note that although additional training improved scores, residents did not feel highly comfortable in performing a cricothyrotomy. This is consistent with previous research demonstrating that the average comfort of graduating residents is at 4.8 on a 10-point scale despite the majority of residents undergoing training on a variety of models [4]. Given the rarity of this procedure, it is unrealistic to expect clinical experience to serve as the sole training method for competence. This makes a strong case to validate and improve this trainer in the future, as it supplements a critically deficient area in EM residency training.

The bleeding cricothyrotomy trainer can also be developed to further enhance fidelity. After data collection, the CAD was modified to reduce membrane size. Additionally, a more flexible filament was used to print the trachea to further increase the fidelity of the trainer. The 3D-printed platform and housing were modified as one of the locking clips initially failed, due to pad thickness exceeding the housing depth. The use of 3D printing in procedural training is a promising area of innovation. Other fields of medicine including neurosurgery, plastic surgery, and cardiovascular surgery utilize 3D printing and simulation for training as well [10]. There are several other high-risk, low-frequency procedures in emergency medicine that are future targets for 3D printed technical trainers.

## Conclusions

This innovative bleeding cricothyrotomy trainer demonstrates structural and functional fidelity. Those with past experience with other models perceived this model to be superior. This trainer also provides a more advanced platform to teach an infrequent yet critical procedural skill to emergency medicine residents.
